# Structural Identifiability of Viscoelastic Mechanical Systems

**DOI:** 10.1371/journal.pone.0086411

**Published:** 2014-02-11

**Authors:** Adam Mahdi, Nicolette Meshkat, Seth Sullivant

**Affiliations:** Department of Mathematics, North Carolina State University, Raleigh, North Carolina, United States of America; Dalhousie University, Canada

## Abstract

We solve the local and global structural identifiability problems for viscoelastic mechanical models represented by networks of springs and dashpots. We propose a very simple characterization of both local and global structural identifiability based on *identifiability tables*, with the purpose of providing a guideline for constructing arbitrarily complex, identifiable spring-dashpot networks. We illustrate how to use our results in a number of examples and point to some applications in cardiovascular modeling.

## Introduction

Mathematical modeling is a prominent tool used to better understand complex mechanical or biological systems [1]. A common problem that arises when developing a model of a biological or mechanical system is that some of its parameters are unknown. This is especially important when those parameters have special meaning but cannot be directly measured. Thus a natural question arises: Can all, or at least some, of the model's parameters be estimated indirectly and *uniquely* from observations of the system's input and output? This is the question of *structural identifiability*. Sometimes the uniqueness holds only within a certain range. In this case, we say that a system is only *locally* structurally identifiable. There are numerous reasons why one would be interested in establishing identifiability. Structural identifiability is a necessary condition for the practical or numerical identifiability problem, which involves parameter estimation with real, and often noisy, data. The unobservable biologically meaningful parameters of a model can only be determined (or approximated) if the model is structurally identifiable. Moreover, optimization schemes cannot be employed reliably since they will find difficulties when trying to estimate unidentifiable parameters [2]. The concept of structural identifiability was introduced for the first time in the work of Bellman and Åström [3]. Since then, numerous techniques have been developed to analyze the identifiability of linear and nonlinear systems with and without controls [2], [4]–[7]; see also [8] for a review of different approaches.

Viscoelastic mechanical models that utilize springs and dashpots in various configurations have been widely used in numerous areas of research including material sciences [9], computer graphics [10], and biomedical engineering to describe mechanical properties of biological systems [11]–[18]. To achieve a desirable response, networks with different numbers of springs and dashpots in various configurations have been constructed. For example, it is well-known that the simplest models of viscoelastic materials such as Voigt (spring and dashpot in parallel) or Maxwell (spring and dashpot in series) do not offer satisfactory representation of the nature of real materials [19]. Thus more complicated configurations are usually constructed and analyzed [17].

In this paper we investigate the identifiability problem of viscoelastic models represented by an arbitrarily complex spring-dashpot network. Although there exist numerous methods that can determine the type of identifiability of a system of ordinary differential equations, generally they are difficult to apply. Our results will show in a remarkably simple way how to verify whether the studied model is (locally or globally) structurally identifiable. In case it is unidentifiable, our method provides an explanation why this is the case and how to reformulate the problem. Moreover, the existing methods usually allow to establish the identifiability only *a posteriori*, i.e. after concrete systems have been established. Thus, we also introduce “identifiability tables”, which allow not only to check but also to construct an arbitrarily complex *identifiable* spring-dashpot network.

### Application to cardiovascular modeling

A particular motivation for this work comes from cardiovascular modeling [21], [22], although the results of this paper can be applied to any viscoelastic modeling approach.

#### Arterial wall

**Figure 1:**
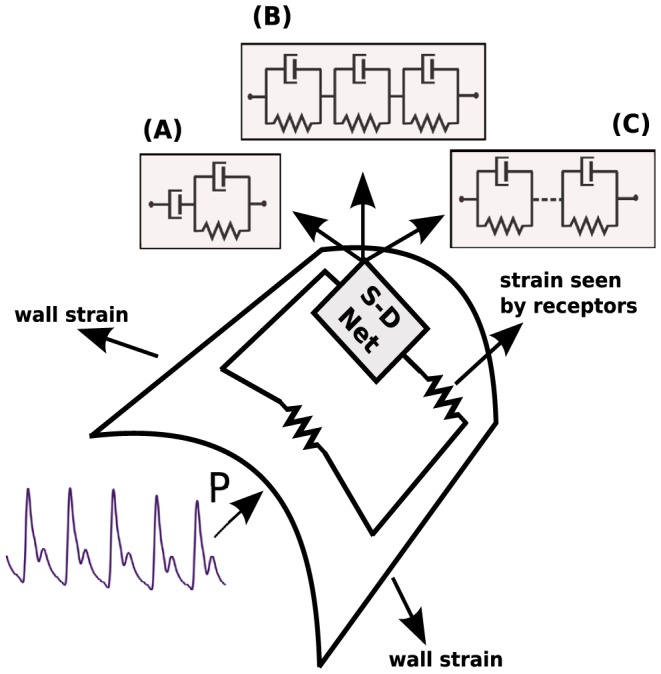
Changing blood pressure (P) causes periodic expansion and contraction of the arterial wall. Spring-dashpot (S-D) networks are often used in order to describe the biomechanical properties of the arterial tissue as well as the strain sensed by various receptors (e.g. baroreceptors) embedded in the arterial wall. Typically a spring (representing a receptor's nerve ending) is combined in series with a S-D network (representing viscoelastic coupling of the nerves to the wall). Recently, several cardiovascular approaches have used the framework described above, in particular, choosing one of the following S-D networks: (A) Burgers-type model [27]; (B) three element Kelvin-Voigt body [20]; (C) generalized Kelvin-Voigt model [21].

Changing blood pressure causes periodic expansion and contraction of the arterial wall (see Fig. 1). It is well-known that the stress-strain curves of the artery walls exhibit hysteresis, which is understood to be a consequence of the fact that the wall is viscoelastic. Another manifestation of the viscoelasticity of the arterial tissue is the stress relaxation experiments under constant stretch (strain). Spring-dashpot (S-D) networks are often used in order to describe the biomechanical properties of the arterial tissue [22]–[24]. Identifiable networks can be determined using the results of this paper (see Theorem 2).

#### Neural activity

It is common to use the spring-dashpot network to describe the neural firing of various sensors (e.g. muscle spindle, baroreceptors), see [20], [25]–[27]. Typically one assumes that the firing activity is proportional to the strain sensed by a spring connected in series with a spring-dashpot network, which represents a local integration of the nerve endings to the arterial wall (see Fig. 1). Then the arterial wall and neural activity models are combined. Although separately each model is structurally identifiable, there is no guarantee that the resulting viscoelastic structure is identifiable. Thus, using our results given in Theorem 5, we can establish whether the combined viscoelastic model is identifiable, and if not, what needs to be modified.

## Results and Discussion

After reviewing basic concepts of viscoelasticity of systems, we present and discuss our main results related to local and global structural identifiability of such systems. Finally, we illustrate our results with a number of examples from the literature.

### Spring-dashpot networks

The ideal linear elastic material follows Hooke's law 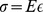, where 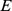 is a Young's modulus (or a spring constant), which describes the relationship between the stress 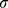 and the strain 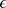. Analogously, the relation 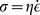 describes the viscous material, where 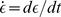 and 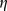 is a viscous constant [28]. In the basic linear viscoelasticity theory, the elastic and viscous elements are combined. In this work, we shall be concerned with the problem of identifiability of networks of springs and dashpots that are essentially one-dimensional. The elements can be combined either in series or in parallel. In order to obtain the relationship between the total stress (force) 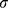 and the total strain (extension) 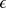 for a given spring-dashpot network, we use two fundamental rules. For two viscoelastic elements connected in series, the stress is the same in both elements, but the total strain is the sum of individual strains on each element. On the other hand, for elements connected in parallel, the strain is the same for both elements, but the total stress is the sum of individual stresses on each element. Now we consider concrete viscoelastic networks, starting with the simplest configurations.

#### Example 1 (Maxwell element)

**Figure 2:**
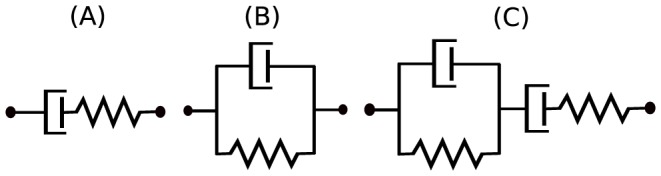
Simple linear viscoelastic models. (A) Maxwell element, (B) Voigt element, (C) Burgers model.

The series combination of a spring, denoted by its constant 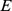, and a dashpot, denoted by its constant 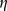, is known as a Maxwell element (see Fig. 2(A)). Since the elements are connected in series, the stress 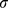 is the same on both elements and the total strain 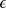 is the sum of strains 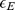 and 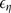 corresponding to the spring and dashpot, respectively. Now, the relationship between the total strain and stress for this system is
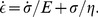
(1)


#### Example 2 (Voigt element)

Another simple example is the Voigt element (also known as Kelvin or Kelvin-Voigt) given in Fig. 2(B). Following the steps outlined in the previous example, we obtain the 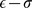 relationship
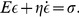
(2)


#### Example 3 (Burgers model)

In our third example we consider a particularly popular four-element model, represented by a Maxwell element combined in series with a Voigt element, and known as the Burgers model (Fig. 2(C)). Denote by subscript 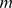 and 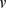 the spring and viscous constants of the Maxwell and Voigt elements, respectively. Note that the stress 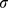 is the same on all three elements connected in series (Voigt, spring and dashpot). Eliminating the corresponding local strains, we obtain the following relationship
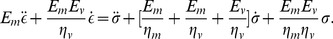
(3)


### Identifiability characterization

First note (cf. Examples 1, 2, and 3) that for any configuration of springs 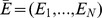 and dashpots 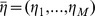, the total strain–stress relationship can always be written as the following 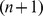-st order linear ordinary differential equation
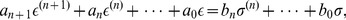
(4)where the coefficients 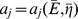 and 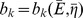 are functions of the spring and dashpot constants. The precise value of 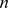 and the forms of 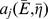 and 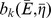 will depend on the particular structure of the spring-dashpot model. Equation 4 is known as the *constitutive equation*. In the context of spring-dashpot networks, identifiability concerns whether or not it is possible to recover the unknown parameters (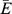 and 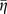) of the system from the governing equation of the model, given only the total stress 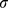 and total strain 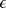. In other words, we assume that we know the stress and the strain at the bounding nodes only and ask if it is possible to determine the unknown parameters (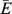 and 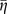). In order to uniquely fix the coefficients of the constitutive equation (4), we require that (4) be *normalized* so that the leading term (in 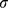 or 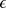, depending on the situation) is monic. Thus, letting the 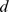 non-monic coefficients of (4) be represented by the vector 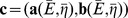, we have the following formal definition of identifiability.

#### Definition 1

Let 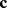 be a function 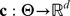, where 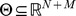 is the parameter space. The model is globally identifiable from 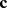 if and only if the map 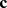 is one-to-one. The model is locally identifiable from 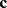 if and only if the map 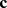 is finite-to-one. The model is unidentifiable from 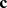 if and only if the map 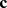 is infinite-to-one.

Note that local identifiability is equivalent to saying that around each point in parameter space there exists a neighborhood on which the function 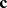 is one-to-one. For example, for the Burgers model considered in Example 3, the coefficient function 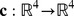 is defined as
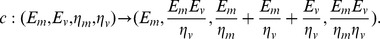
Technically speaking, in this paper we will consider the slightly weaker notion of *generic global identifiability* (or generic local identifiability, or generic unidentifiability), where *generic* means that the property holds almost everywhere. We will omit the use of the term generic when speaking of identifiability.

Definition 1 implies that if there are more parameters than non-monic coefficients, then the system must be unidentifiable. Thus, a necessary condition for structural identifiability is that the number of parameters 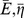 (elements of the network) is less than or equal to the number of non-monic coefficients in the constitutive equation (4). We will soon show that the number of non-monic coefficients is bounded by the number of parameters in spring-dashpot networks. Thus, in this case, a necessary condition for structural identifiability is that the number of parameters and non-monic coefficients are equal. We will prove that, remarkably, in the case of viscoelastic models represented by a spring-dashpot network, the converse to this statement holds as well.

#### Theorem 2 (Local identifiability)

*A viscoelastic model represented by a spring-dashpot network is locally identifiable if and only if the number of non-monic coefficients of the corresponding constitutive* *equation (4)* *equals the total number of its moduli* 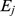 *and viscosity parameters* 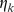 *.*

Note that although the constitutive equation (4) is a linear differential equation, its coefficients considered as functions of spring and viscous constants are not linear functions of the parameters (see (3)). Thus, Theorem 2 allows to reduce the difficult problem of checking one-to-one or finite-to-one behavior of nonlinear functions to simply counting the number of parameters (springs and dashpots) and non-monic coefficients of the constitutive equation and asking whether the two numbers are equal. The positive answer implies local identifiability, whereas a negative answer implies unidentifiability. Consider, for example, the Maxwell and Voigt elements, and the Burgers model. We note that the constitutive equations (1), (2), and (3) for all three models are already in the normalized form. Now, simply by counting the number of parameters and the non-monic coefficients of the constitutive equations, we see that the two are equal for each model. Thus, by the above theorem, all three models are locally structurally identifiable.

### Constructing identifiable models

Now we examine when combining two identifiable models results also in an identifiable model. This will allow us to construct arbitrarily complex and identifiable spring-dashpot networks.

We start with an observation, which we prove in the following section, related to the possible form of any differential equation that describes a spring-dashpot network.

#### Proposition 3

*Every spring-dashpot network, given by* *equation (4)* *, has one of the four possible types*

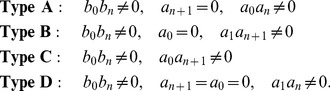
(5)


Recall that 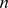 is the highest derivative of the stress component 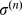, which appears in the total strain-stress equation (4). Now we illustrate the different types of networks defined in the above proposition by considering the simplest elements.

#### Example 4

For a spring, given by 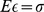, we have 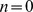 (only 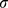 appears in the constitutive equation, but none of its derivatives), 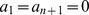, and 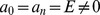. Therefore a spring is of type A. Note that for a dashpot, which is given by 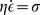, we also have 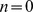 but 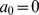 and 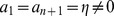. Thus, according to notation given in Proposition 3, a dashpot is of type B. For the Voigt element, given by (2), we have 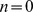 as well as 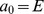 and 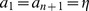 (that is 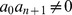). We conclude that it is of type C. Finally, a Maxwell element is given by (1). Note that here 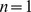 (since 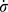 appears in (1)), 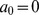, 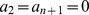, and 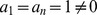. Thus a Maxwell element is of type D.

Once the constitutive equation has been determined for a given spring-dashpot network, it is very easy to establish the type that it belongs to. Unfortunately, 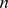 does not always have a physical significance. The value of 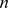 is determined by the specific network and cannot be easily related to the number of springs and dashpots as we will illustrate later on.

#### Theorem 5 (Local identifiability)

*Consider two locally identifiable spring-dashpot systems* 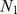 *and* 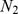 *of one of the four types* 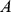 *,* 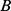 *,* 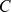 *,* 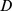 *. Then the new model resulting in joining* 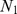 *and* 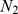 *either in parallel or in series is of the type indicated by the Identifiability* *Tables 1* *and* *2* *. The letter u indicates that the network is unidentifiable, otherwise it is identifiable of the given type.*

**Table 1:** Identifiability Tables: Parallel connection.

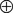	A	B	C	D	u
**A**	u	C	u	A	u
**B**	C	u	u	B	u
**C**	u	u	u	C	u
**D**	A	B	C	D	u
**u**	u	u	u	u	u

When connecting two identifiable spring-dashpot networks of one of the types 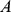, 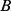, 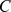, 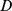, or an unidentifiable 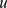 in series the above tables establish the type of the resulting identifiable system. If the resulting structure is unidentifiable it is indicated by 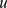.

**Table 2:** Identifiability Tables: Series connection.

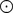	A	B	C	D	u
**A**	u	D	A	u	u
**B**	D	u	B	u	u
**C**	A	B	C	D	u
**D**	u	u	D	u	u
**u**	u	u	u	u	u

When connecting two identifiable spring-dashpot networks of one of the types 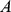, 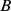, 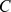, 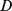, or an unidentifiable 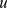 in parallel, the above tables establish the type of the resulting identifiable system. If the resulting structure is unidentifiable it is indicated by 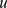.

There are several ways one could use the above theorem. One way is to establish the local identifiability of a given spring-dashpot network. Contrary to our similar result given in Theorem 2, this can be done without actually calculating the constitutive equation. We will show how to apply Theorem 5 to establish structural identifiability after first introducing some notation. Given any two spring-dashpot models 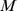 and 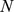, we use the following notation 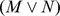 and 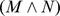 to denote respectively the parallel and series combination of 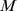 and 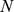. Let 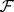 denote the function that takes a spring and dashpot model 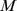 and outputs its type (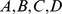) if it is locally identifiable, and 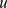 if it is unidentifiable. To apply 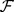 to a complicated model built up from springs and dashpots using series and parallel connections, we replace any springs and dashpots with their respective types 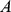 and 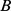 as well as the operations 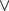 and 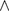 with 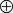 and 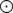, respectively. Then we apply the operations in the Identifiability Tables 1 and 2.

#### Example 4 (Local identifiability of the Maxwell element)

Note that the Maxwell model shown in Fig. 2(A) can be symbolically written as
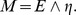
In this formula, we simply replace the spring and the dashpot with 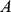 and 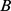, respectively, as well as the operations 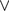 and 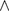 with 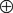 and 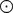, respectively, to obtain
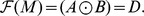
Thus we conclude that the Maxwell model is locally identifiable and is of type 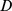.

#### Example 5 (Local identifiability of the Burgers model)

Similarly, the Burgers model shown in Fig. 2(C) can be symbolically written as
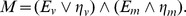
To check the local identifiability, we find 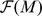 and use Tables 1 and 2 to obtain
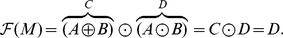
We conclude that the Burgers model is locally identifiable and of type 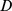.

In the next example we show how we can easily establish local structural identifiability of a more complicated network.

#### Example 6 (Dietrich et al. [19])

**Figure 3:**
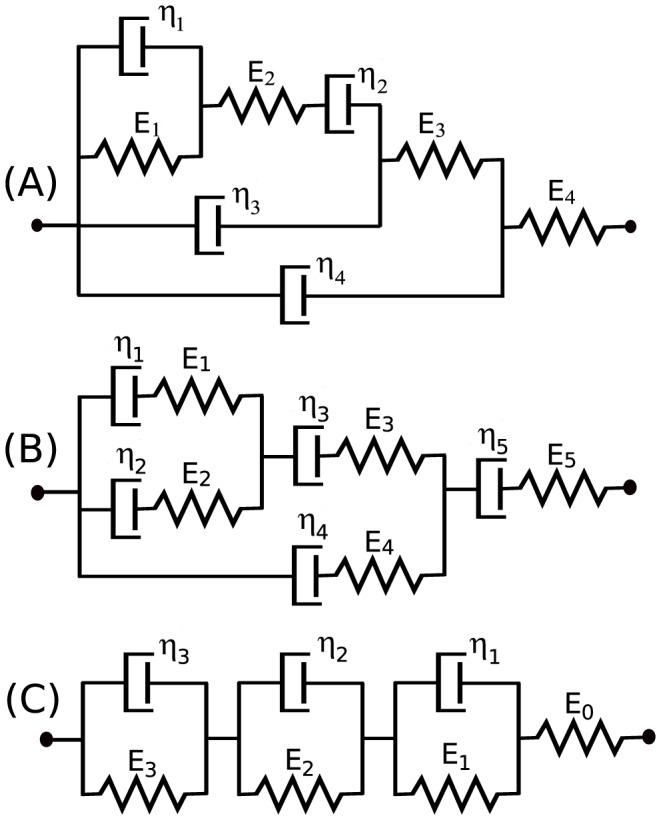
(A) Multi-parameter linear viscoelastic model considered by Dietrich et al. [19]. (B) Ten element viscoelastic model studied in [13], (C) A viscoelastic model of used to describe the baroreceptor nerve ending coupling to the arterial wall (see [20] and [21], [29]).

Consider a viscoelastic material studied in [19] and represented by a spring-dashpot network shown in Fig. 3(A). It can be symbolically represented by
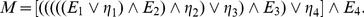
(6)Again, we can verify the local identifiability of the above model using Tables 1 and 2 and obtain
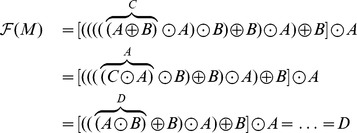
This simple computation confirms that the model is locally structurally identifiable.

Our method can also verify if a network is unidentifiable, providing the reason for the lack of its identifiability. Consider the following example.

#### Example 7 (Unidentifiable model)

Consider a viscoelastic model used in [13] and shown in Fig. 3(B). Using the notation previously introduced, it can symbolically be written as
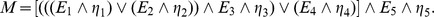
Now applying Tables 1 and 2, we obtain
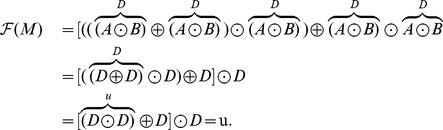
Whenever Tables 1 and 2 indicate 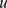 (i.e. the corresponding substructure is unidentifiable), this inevitably leads to the whole model being unidentifiable. Moreover, our method can also explain what is the reason for the lack of identifiability. In this example the situation is simple: joining in series a Maxwell element (type D) with a generalized Maxwell model leads to an unidentifiable network.

So far we have considered only *local* identifiability of mechanical systems. Now we complete the presentation and discussion of our results by introducing a criterium, which establishes when a given network is *globally* structurally identifiable.

#### Theorem 6 (Global identifiability)

*A viscoelastic model represented by a spring-dashpot network is globally identifiable if and only if it is locally identifiable and the network is constructed by adding either in parallel or in series at the bounding nodes exactly one basic element (spring or dashpot) at a time.*

Note that the network given in Fig. 3(A) and considered in Example 6 was deemed locally structurally identifiable. We note that it can be constructed by adding just one element at a time and therefore it is *globally* structurally identifiable. Similarly, all the simple models shown in Fig. 2 can also by constructed adding only one element at a time, and since they are locally identifiable, we conclude that they are also globally structurally identifiable. Now consider a model which is locally, but not globally, structurally identifiable.

#### Example 8 (Local but not global identifiability)

Consider a generalized Kelvin-Voigt model shown Fig. 3(C) and used in [20], [29]) in the context of cardiovascular modeling. It can be symbolically represented by
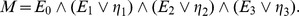
Thus the local identifiability can be checked by computing
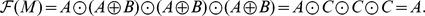
We immediately conclude that the network is locally identifiable. In order to verify whether it is also globally identifiable, note that this network *cannot* be constructed by adding only one element at a time. Thus the system is only locally, but not globally, identifiable. However, in this case the non-global identifiability arises from merely permuting the parameters among the three Voigt elements.

## Analysis

In this section, we prove the main results from the previous section. To do this requires a careful analysis of the structure of the constitutive equation after combining a pair of systems in series or in parallel.

Let 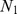 and 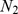 be spring-dashpot models whose respective constitutive equations are 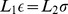 and 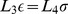, where 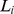 represent linear differential operators. We can write the differential operators (in general form) as:
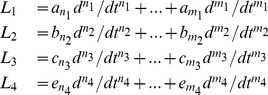
(7)


*Remark*. Table 3 shows that there are restrictions on the values of the 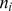 and 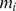, e.g. the differential order of the lowest order term in 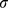 is always zero and the differential order of the lowest order term in 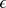 is zero or one, but we leave the operators in general form for simplicity.

**Table 3:** Possible types of constitutive equations.

Type	Shape in 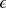	Shape in 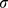
A	[ 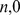 ]	[ 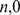 ]
B	[ 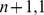 ]	[ 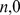 ]
C	[ 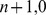 ]	[ 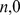 ]
D	[ 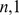 ]	[ 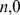 ]

The four possible types of constitutive equations, defined by the shapes of the linear operators acting on 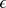 and 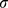, written in brackets.

We now show the form of the resulting constitutive equation after combining these systems in series or in parallel, in terms of these differential operators. In what follows, we will treat the differential operators 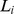 as polynomial functions in the variable 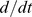. For example, 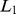 can be thought of as a polynomial 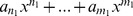.

### Series connection

Suppose that 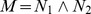 is a series connection of models 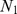 and 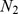, whose constitutive equations are 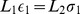 and 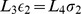, respectively. Then the stresses (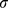) are the same for the two systems while the strains (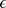) are added. If 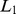 and 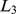 are relatively prime, then the constitutive equation of 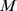 is:
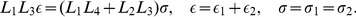
(8)We assume that 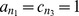, so that the constitutive equation is monic. If 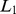 and 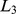 have a common factor, then the constitutive equation of 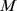 is obtained by dividing (8) by the greatest common divisor of 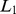 and 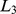.

### Parallel connection

Suppose that 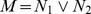 is a parallel connection of models 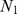 and 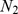, whose constitutive equations are 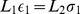 and 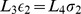, respectively. Then the strains (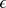) are the same for the two systems while the stresses (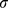) are added. If 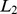 and 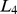 are relatively prime, then the constitutive equation is:
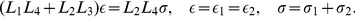
(9)We assume that 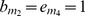, so that the constitutive equation is monic. If 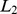 and 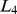 have a common factor, then the constitutive equation is obtained by dividing (9) by the greatest common divisor of 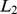 and 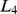.

### Types of networks

Now we prove Proposition 3, that is, we show that every spring-dashpot network, given by equation (4), has one of the four possible types displayed in Table 3, which are defined by the *shapes* of the linear operators 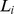 acting on 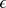 and 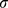. We make this notion precise:

#### Definition 7

The *shape* of a linear operator 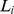 is a pair of numbers, written 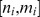, where 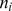 is the highest differential order and 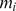 is the lowest different order.

We note that a spring is of type A and a dashpot is of type B. A Voigt element is formed by a parallel extension of types A and B, which forms type C, and a Maxwell element is formed by a series extension of types A and B, which forms type D. The properties of these four types are displayed in Table 3. We can now form the 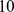 possible combinations of pairing two of these types in series and the 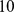 possible combinations of pairing two of these types in parallel. In Tables 4 and 5, we show the 20 total possibilities and demonstrate that each pairing results in a type A, B, C, or D. Since every spring-dashpot network can be written as a combination, in series or in parallel, of springs and dashpots, then we have shown by induction that joining any two spring-dashpot networks in series or in parallel results in one of these four types.

**Table 4:** Series connection.

Type	Shape in 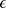	Shape in 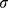	Non-monic coefficients	Parameters	Identifiable?	Type
(A,A)	[ 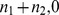 ]	[ 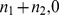 ]	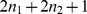	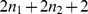	Not Id	A
(A,B)	[ 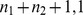 ]	[ 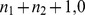 ]	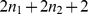	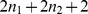	Id	D
(A,C)	[ 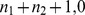 ]	[ 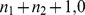 ]	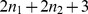	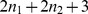	Id	A
(A,D)	[ 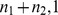 ]	[ 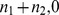 ]	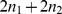	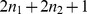	Not Id	D
(B,B)	[ 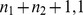 ]	[ 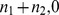 ]	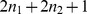	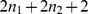	Not Id	B
(B,C)	[ 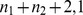 ]	[ 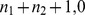 ]	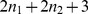	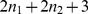	Id	B
(B,D)	[ 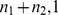 ]	[ 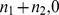 ]	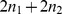	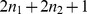	Not Id	D
(C,C)	[ 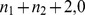 ]	[ 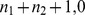 ]	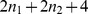	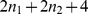	Id	C
(C,D)	[ 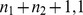 ]	[ 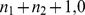 ]	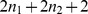	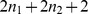	Id	D
(D,D)	[ 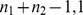 ]	[ 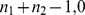 ]	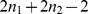	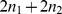	Not Id	D

Two systems of types A, B, C, or D are combined in series, where in the first system 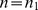 and in the second system 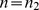.

**Table 5:** Parallel connection.

Type	Shape in 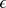	Shape in 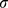	Non-monic coefficients	Parameters	Identifiable?	Type
(A,A)	[ 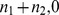 ]	[ 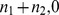 ]	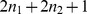	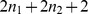	Not Id	A
(A,B)	[ 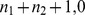 ]	[ 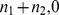 ]	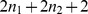	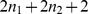	Id	C
(A,C)	[ 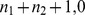 ]	[ 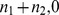 ]	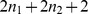	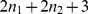	Not Id	C
(A,D)	[ 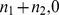 ]	[ 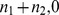 ]	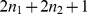	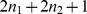	Id	A
(B,B)	[ 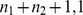 ]	[ 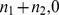 ]	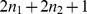	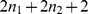	Not Id	B
(B,C)	[ 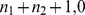 ]	[ 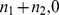 ]	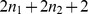	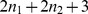	Not Id	C
(B,D)	[ 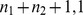 ]	[ 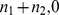 ]	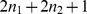	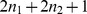	Id	B
(C,C)	[ 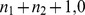 ]	[ 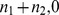 ]	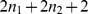	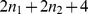	Not Id	C
(C,D)	[ 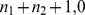 ]	[ 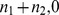 ]	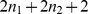	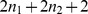	Id	C
(D,D)	[ 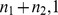 ]	[ 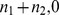 ]	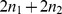	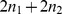	Id	D

Two systems of types A, B, C, or D are combined in parallel, where in the first system 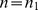 and in the second system 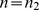.

*Remark*. We note that if a type B or D is combined in series with a type B or D, then 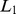 and 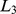 have a common factor (since both lacked a constant term), so the equation 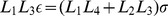 is divided by 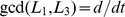 to arrive at the shapes listed in the table.

In addition to the type of equation that results after combining two equations of types 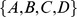, we have in Tables 4 and 5 the resulting identifiability properties of each equation, which we will obtain in the next section. Note that Definition 1 implies that if there are more parameters than non-monic coefficients, then the system must be unidentifiable. The tables show that the number of non-monic coefficients is bounded by the number of parameters, thus a necessary condition for identifiability is that the number of parameters equals the number of non-monic coefficients in the constitutive equation (4). In the next section, we show that this is also a sufficient condition.

### Local identifiability

Consider a spring-dashpot system 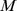 whose final step connection is a series connection of two systems 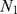 and 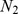, i.e. 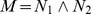. Since the number of non-monic coefficients in any spring-dashpot model is always less than or equal to the number of parameters in that model, we know that a necessary condition for this system to be locally identifiable is that 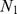 and 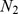 are both locally identifiable. Let 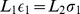 be the constitutive equation for 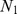 and 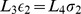 be the constitutive equation for 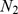. Each of the operators 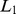, 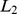, 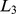, and 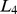 will have a fixed shape determined by the structure of 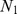 and 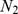. Assuming that 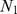 and 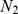 are locally identifiable, we can choose parameters in each of the models 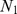 and 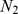 so that the coefficients of these constitutive equations are arbitrary numbers. Thus, deciding identifiability of this system amounts to determining whether the map that takes the pair of equations 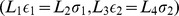 to the constitutive equation 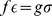, where 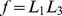, 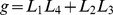, 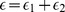, and 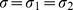 (cf. (8)), for the system 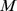 is finite-to-one or not.

The same reasoning works *mutatis mutandis* for parallel connections, where we now concern ourselves with the map from the pair of equations 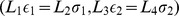 with generic coefficients to the constitutive equation for 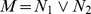 given in (9).

To make the above, intuitive, statements precise we introduce the following definition.

#### Definition 8

The *shape factorization problem* for a quadruple of shapes
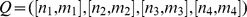
is the following problem: for a generic pair of polynomials 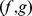 with 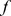 monic such that the 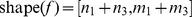 and 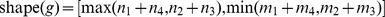, do there exist finitely many quadruples of polynomials 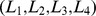 with 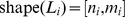, 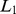 and 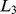 are monic, and such that 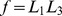 and 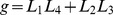
? A quadruple of shapes 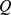 is said to be *good* if the shape factorization problem for that quadruple has a positive solution.

Since the above definition introduces one of the key concepts of the paper, in the following example we shall further illustrate the meaning of the shape factorization problem.

#### Example 9

Suppose that our quadruple
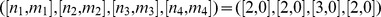
which is a special case of joining models of types 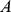 and 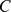 in series. The shape factorization problem in this case asks the following question:

Let 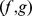 be a generic pair of polynomials where 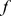 and 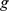 are degree 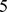 polynomials with nonzero constant term and 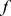 is monic:
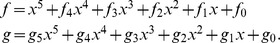
Do there exist finitely many polynomials
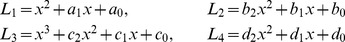
such that 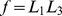 and 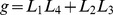
? Or to say it another way, for generic values of 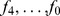 and 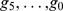, does the system of 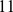 equations in 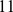 unknowns:
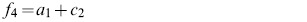


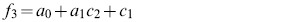


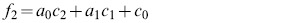


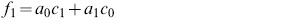


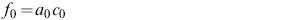


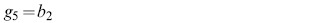


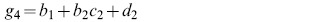


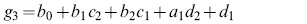


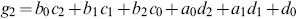


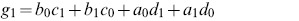


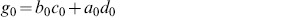
have only finitely many solutions?

The language of shape factorization problems and the remarks in the preceding paragraphs allow us to reduce the local identifiability problem for a spring-dashpot system to determining whether a certain quadruple is a good quadruple.

#### Proposition 10

*Let* 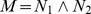 *be a spring-dashpot model joined in series from* 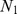 *and* 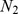 *, where* 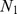 *has constitutive equation* 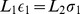 *of shapes* 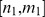 *and* 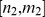 *, respectively, and* 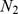 *has constitutive equation* 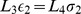 *of shapes* 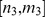 *and* 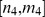 *, respectively. Then the model* 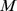 *is locally identifiable if and only if*

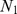
 *and* 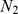 *are locally identifiable, and*

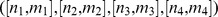
 *is a good quadruple*.

*Similarly, if* 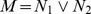 *is a spring-dashpot model joined in parallel from* 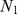 *and* 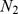 *, then* 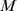 *is locally identifiable if and only if*

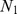
 *and* 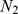 *are locally identifiable, and*

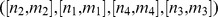
 *is a good quadruple.*

So what remains to show is that, for the shapes that arise in spring-dashpot models, whether a quadruple of shapes is a good quadruple only depends on the types (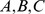, or 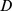) of the systems being combined. The proof of this statement will occupy the rest of this section.

Let 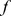 and 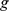 be two polynomials. Note that for given fixed shapes, 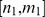 and 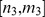, there are at most finitely many factorizations 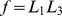, where 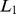 has shape 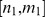 and 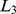 has shape 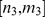 and both are monic. This is because there are at most finitely many ways to factorize a monic polynomial into monic factors. Once we fix one of these finitely many choices for 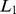 and 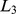, the equation 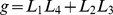 is a linear system in the (unknown) coefficients of 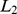 and 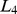.

For a polynomial 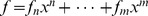 of shape 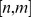, we can write the coefficients of 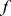 as a vector, which we denote
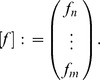
Let 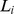 have shape 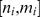, as defined in Equation (7). The vector of coefficients of 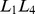 can be written as the result of a matrix vector product as:
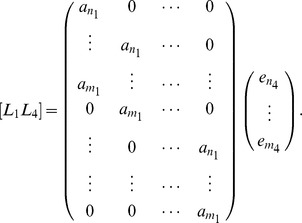
We will refer to this product as 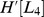, where 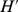 is a 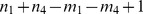 by 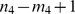 matrix. Likewise, the coefficients of 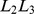 can be written as the result of a matrix vector product as:
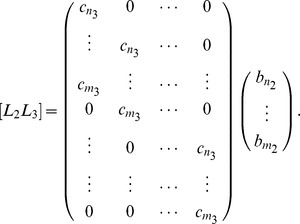
We will refer to this product as 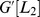, where 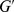 is a 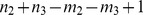 by 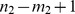 matrix. Then we call the *matrix factored form* of 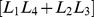 the expression:
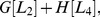
(10)where the matrices 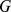 and 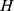 are the matrices 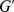 and 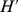 padded with rows of zeros so that coefficients corresponding to monomials of the same degree appear in the same row. This makes 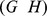 a 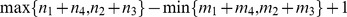 by 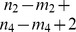 matrix.

We can now state a criteria for determining if the shape factorization problem has finitely many solutions:

#### Proposition 11

*The quadruple* 
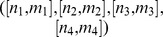
 *is a good quadruple if and only if the matrix* 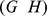 *is generically invertible.*

*Proof.* We can write the shape factorization problem of type 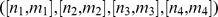 in matrix factored form as 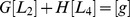 (see (10)), so that
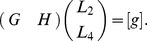
This system has a unique solution if and only if 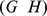 is generically invertible, i.e. invertible for a generic choice of parameter values.

#### Example 12

Suppose that our quadruple 
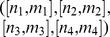
 is 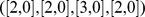, which is a special case of joining models of types 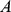 and 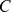 in series. The resulting matrix 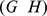 is the matrix
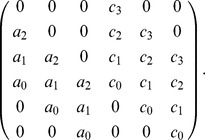



We now determine when this matrix 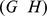 is generically invertible, i.e. square and full rank. The *Sylvester matrix* associated to two polynomials 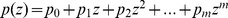 and 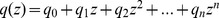 is the 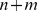 by 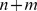 matrix that has the coefficients of 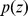 repeated 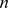 times as columns and the coefficients of 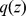 repeated 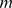 times as columns in the following way:
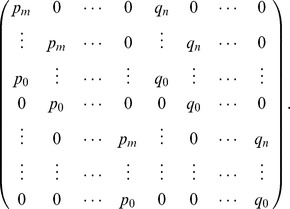


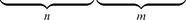
The determinant of the Sylvester matrix of the two polynomials 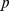 and 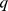 is the *resultant*, which is zero if and only if the two polynomials have a common root. In particular, for generic polynomials 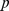 and 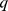, the Sylvester matrix is invertible [30, Chapter 3].

We will use the Sylvester matrix in the following way. We will show that there are submatrices of 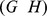 that correspond to the Sylvester matrix associated to 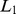 and 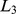.

#### Proposition 13

*If the matrix* 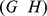 *is square, then it is generically invertible.*

*Proof.* We claim that the columns of 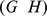 can be ordered so that the resulting matrix has the shape
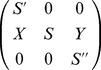
(11)where 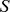 is the Sylvester matrix associated to the nonzero coefficients of 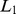 and 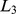. Note that this means that we might shift the coefficients down if necessary so there are no extraneous zero terms of low degree (i.e. if the shape is 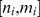 with 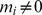). The matrix 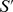 is a square lower triangular matrix with nonzero entries on the diagonal, and 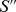 is a square upper triangular matrix with nonzero entries on the diagonal. This will prove that 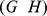 is invertible, since its determinant will be the product of the determinants of 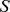, 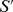 and 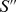, all of which are nonzero. To prove that claim requires a careful case analysis.

The number of columns of 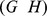 is 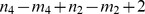 and the number of rows is 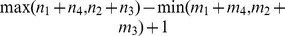. Without loss of generality, we can assume that the maximum is attained by 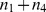. We need to distinguish between the two cases where the minimum is attained by 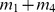 and by 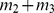.

**Case 1:** 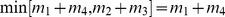 **.** Since 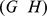 is a square matrix, this implies that 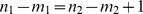. In this case we group the columns of 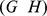 in the following order.

1. The first 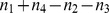 columns of 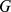

2. Then the next 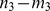 columns of 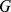

3. Then all 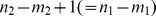 columns of 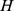

4. Then the remaining 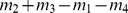 columns of 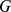.

This choice has the property that the middle two blocks of columns together have the desired form, since we have chosen to start including columns from 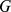 and 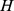 precisely when they both have nonzero entries in the same rows, and stopping the formation of these when they stop having nonzero entries in the same rows, which has the correct form. Note we have used all columns of 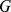 since
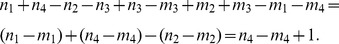



**Case 2:** 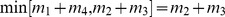 **.** Note that since 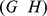 is square, this implies that 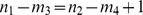. In this case, we do not need to reorder the columns to obtain the desired form.

We mention how to block the columns to obtain the desired form.

1. The first 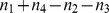 columns of 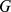

2. Then the next 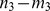 columns of 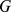

3. Then the first 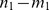 columns of 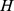

4. Then the remaining 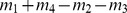 columns of 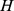.

Note that we have the desired number of columns from the second and third blocks, and we have chosen them so that that those columns have nonzero entries at exactly the same rows. Furthermore, we have used all columns of 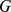 since
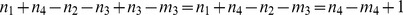
and all columns of 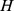 since
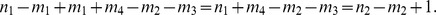



#### Example 14

We can rewrite the matrix in Example 12 as
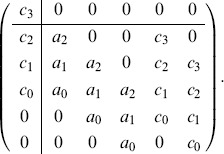
Here the the 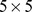 matrix in the lower righthand corner is the Sylvester matrix, the matrix 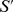 is the 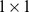 matrix in the upper lefthand corner, and the matrix 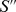 is the empty matrix.

*Proof of Theorem 2.* We will show that if the number of parameters equals the number of non-monic coefficients, then the matrix 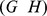 is square. By Propositions 11 and 13, this will imply that the model is locally identifiable.

Let 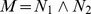 be a spring-dashpot model joined in series from 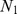 and 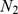, where 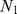 has constitutive equation 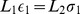 of shapes 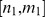 and 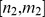, respectively, and 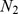 has constitutive equation 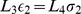 of shapes 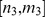 and 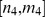, respectively. By induction, we can assume that the number of parameters equals the number of non-monic coefficients for the systems 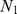 and 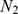, i.e. there are 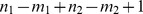 parameters in the first and 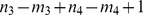 in the second. Assume the number of parameters equals the number of non-monic coefficients in this full system, i.e.
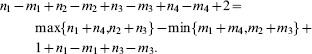
Subtracting 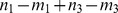 from both sides, we get that
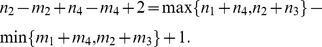
From the definition of 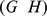, this means the number of rows equals the number of columns, so that 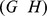 is square.

The argument for the parallel extension is identical and is omitted.

*Proof of Theorem 5.* Theorem 2 shows that the model is locally identifiable if and only if the number of parameters equals the number of non-monic coefficients. Thus the identifiability properties of the 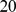 cases in Tables 4 and 5 are determined by checking if the numbers in the columns corresponding to the number of parameters and the number of non-monic coefficients are equal.

### Global identifiability

We now determine necessary and sufficient conditions for global identifiability.

#### Proposition 15

*Let* 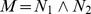 *be a spring-dashpot model joined in series from* 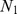 *and* 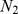 *, where* 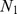 *has constitutive equation* 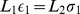 *of shapes* 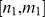 *and* 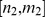 *, respectively, and* 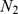 *has constitutive equation* 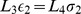 *of shapes* 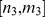 *and* 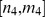 *, respectively. Then the model* 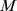 *is globally identifiable if and only if*

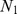
 *and* 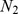 *are globally identifiable,*
2. *The shape factorization problem for the quadruple* 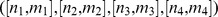 *generically has a unique solution.*

*Similarly, if* 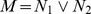 *is a spring-dashpot model joined in parallel from* 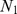 *and* 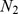 *, then* 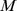 *is globally identifiable if and only if*

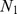
 *and* 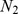 *are globally identifiable, and*
2. *The shape factorization problem for the quadruple* 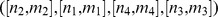 *generically has a unique solution.*

*Proof.* We handle the case of series extensions, parallel extensions being identical. Let 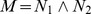. Clearly, 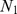 and 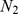 must be globally identifiable otherwise we could give two sets of parameters yielding the same constitutive equation for 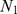, which could then be combined with parameters for 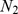 to get two sets of parameters for 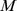 yielding the same constitutive equation.

Now if the shape factorization problem has a unique solution, there is a unique way to take the constitutive equation for 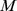 and solve for the constitutive equations for 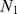 and 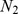, since 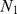 and 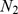 are globally identifiable, there is a unique way to solve for parameters of those models giving a unique solution for parameters for 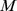. Conversely, if there were multiple solutions to the shape factorization problem, then by global identifiability of 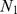 and 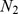, we could solve all the way back to get multiple parameter choices for the same parameter choice for 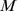.

Note that in our analysis of the shape factorization problem in the previous section, we saw that once 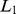 and 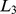 are chosen among all their finitely many values, when the model is locally identifiable there is a unique way to then construct 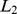 and 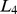. Hence, the shape factorization problem has a unique solution when there is a unique way to factor 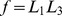. This happens if and only if either 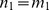 or 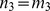, otherwise, generically, we can exchange roots of 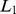 and 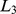 giving multiple solutions.

#### Corollary 16

*Suppose that* 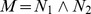 *is globally identifiable. Then either* 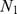 *or* 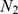 *must have been one of a spring, a dashpot, or a Maxwell model. Suppose that* 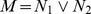 *is globally identifiable. Then either* 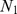 *or* 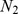 *must have been one of a spring, a dashpot, or a Voigt model.*

*Proof.* The four models given by the spring, dashpot, Voigt, and Maxwell elements are the only four locally identifiable models that have the property that at least one of the differential operators in its constitutive equation has exactly one term. This can be seen by analyzing the four types (A,B,C,D) and looking at all possibilities that arise on combining two equations. Once both operators do not have a single term, no model combined from such a model can have an operator with a single term.

The three choices for the series connection (spring, a dashpot, or a Maxwell model) are the three of four models that put a differential operator with a single term in the correct place so there could be a unique solution to the shape factorization probelm. Similarly for the parallel connection.

*Proof of Theorem 6.* Clearly a globally identifiable model is locally identifiable. By Corollary 16, we must be able to construct such a globally identifiable model by adding at each step either a spring, dashpot, Maxwell or Voigt element at each step, but when adding a Maxwell element it must be used in series and when using a Voigt element it must have been added in parallel. However, adding a Maxwell element in series can be achieved by adding a spring and then a dashpot both in series. Similarly, adding a Voigt element in parallel can be achieved by adding a spring and then a dashpot both in parallel. Hence, we can work only adding springs or dashpots at each step.
